# Incidence, Risk Factors, and Subsequent Health Outcomes of Pyogenic Liver Abscesses: A Scoping Review of Evidence From Population-Based Studies

**DOI:** 10.1155/grp/3915024

**Published:** 2025-09-23

**Authors:** Shiwen Wang, Zhihui Chang

**Affiliations:** ^1^China Medical University, Shenyang, Liaoning, China; ^2^Department of Radiology, Shengjing Hospital of China Medical University, Shenyang, Liaoning, China

**Keywords:** incidence, population-based study, pyogenic liver abscess, risk factor, subsequent health outcome

## Abstract

**Background:** Pyogenic liver abscess (PLA) is a critical infectious disease with varying incidence across global regions. There is a growing body of large-scale, population-based studies that offer a comprehensive understanding of the disease.

**Objective:** To allow clinicians to gain a more comprehensive and systematic understanding of PLA, this review of the published literature was conducted to summarize the incidence of PLA and identify comprehensive risk factors and subsequent health outcomes.

**Methods:** To obtain more reliable and convincing data, we searched through the electronic databases PubMed, Web of Science, and the China National Knowledge Infrastructure (CNKI), using the following search terms: pyogenic liver abscess and population-based study. The initial search was executed on 26 December 2022 and subsequently updated on 30 May 2025.

**Results:** The search identified 43 eligible studies for the final analyses. Among the 43 studies, 11 included the incidence of PLA, 21 included information on the risk factors, and 11 included the prognosis of PLA. According to the distribution of study locations, most of the studies were from Taiwan, China, which had the highest incidence in the world, reaching 17.59 per 100,000. The results highlight that the risk factors for PLA encompass liver cirrhosis, hepatobiliary malignancy, liver transplantation, biliary disease, and diabetes mellitus. Furthermore, we observed that PLA increased the risk of subsequent health complications, including gastrointestinal tumors and infection.

**Conclusion:** The increasing prevalence and multifaceted implications of PLA underscore the imperative for medical professionals to remain updated on its epidemiology, risk factors, and subsequent health outcomes. Such awareness is pivotal for effective community prevention, clinical intervention, and long-term patient management.

## 1. Introduction

Pyogenic liver abscess (PLA) is a critical infectious condition, characterized by the invasion of pathogenic bacteria into the liver. In recent years, *Klebsiella pneumoniae* liver abscess (KPLA) caused by hypervirulent *Klebsiella pneumoniae* (hvKP) has a significant increasing trend in incidence worldwide [1, 2], especially in Asia [3]. According to Asian epidemiological data, it was observed that the annual increase in incidence was 0.86 cases per 100,000 population from 1996 to 2004 [4]. Despite its global prevalence, the intricacies of PLA remain shrouded in complexity, with factors influencing its onset being multifaceted and early clinical signs often eluding clear identification. Historically, the majority of research on PLA has been limited to single-center studies with small sample sizes. Such studies, while valuable, have led to a fragmented understanding of PLA, with data often varying across different geographical regions and medical institutions.

However, the landscape of PLA research has witnessed a transformative shift in recent years. The emergence of large-scale, population-based studies has provided a more comprehensive understanding of the disease, addressing many gaps left by previous research endeavors. These comprehensive studies not only have enriched our understanding of the epidemiology of PLA but have also shed light on its predominant risk factors and subsequent health implications.

Given the profound clinical significance of PLA, there is an urgent need to consolidate current evidence, offering clinicians a cohesive overview of the disease. This review seeks to fulfill that need, aiming to provide a comprehensive synthesis of the latest developments in PLA research. Through this comprehensive analysis, we hope to inform and guide community-based prevention strategies, clinical interventions, and posttreatment care, ultimately contributing to better patient outcomes.

## 2. Methods

### 2.1. Identification of Research Question

The primary objective of this review is to explore the worldwide differences in the incidence of PLA, the factors that increase risk, and the impact on subsequent health. To address these aims, we formulated the following research questions:

1. What is the global incidence of PLA, and are there significant geographical variations?
2. What are the established risk factors for PLA development?
3. What are the prognostic outcomes and subsequent health consequences of PLA?

### 2.2. Identification of Relevant Studies

This review was meticulously carried out in alignment with the PRISMA-ScR checklist guidelines [5]. To ensure the acquisition of robust and credible data, we embarked on a comprehensive search of the PubMed, Web of Science, and the China National Knowledge Infrastructure (CNKI, https://www.cnki.net/), employing the specific search terms for PubMed and Web of Science: “pyogenic liver abscess” and “population-based study.” For the CNKI, the main subject terms, “pyogenic liver abscess” and “population-based study” with a “core journal” filter were searched. The initial search was executed on 26 December 2022 and subsequently updated on 30 May 2025.

The inclusion criteria are as follows:

a. Population-based studies investigating risk factors and subsequent health complications, with a minimum sample size of 2000 and available data on PLA incidence;
b. The subsequent health effects must be evaluated after a PLA was present for a while.

All studies meeting these criteria were included.

The exclusion criteria are as follows:

a. Studies with a sample size of less than 2000 regarding risk factors and subsequent health complications;
b. Articles lacking data on incidence, risk factors, or subsequent health effects.

Studies meeting either criterion were excluded.

### 2.3. Study Selection

Articles retrieved from the PubMed, Web of Science, and CNKI databases and meeting the criteria were downloaded, after deletion of duplicates, for primary screening at the title and abstract level based on previously scheduled questions and criteria. The full text was screened by one author and then verified by another, with any disagreements resolved through consensus.

### 2.4. Charting of Obtained Data

Articles selected for inclusion underwent meticulous screening for pertinent outcome data. Following this screening, we devised a table comprising six essential elements: study, country or region, year of study, study population size, incidence, and risk factors. In addition, another table and an illustrative figure were developed to elucidate the subsequent health implications of PLA. To offer a clear visual representation, the distribution of each study and the incidence of PLA were portrayed using a pie chart and a world map, respectively. Finally, the unit used in this study to calculate the incidence of PLA is uniformly cases per 100,000 people.

## 3. Results

**Figure 1:**
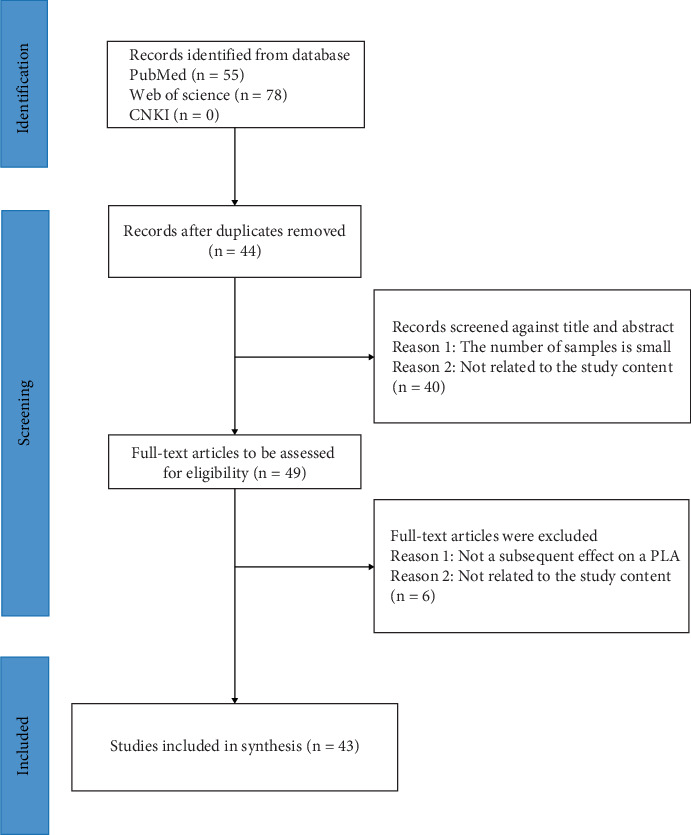
Flowchart depicting the process of study selection and inclusion.

**Figure 2:**
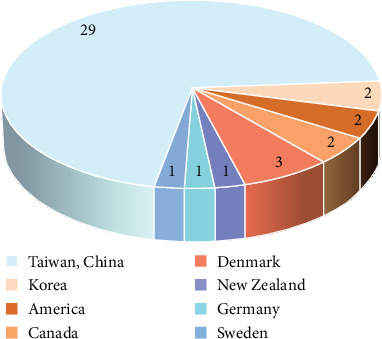
Pie chart illustrating the distribution of countries and regions represented in the reviewed literature.

Initially, 133 articles were retrieved from the database. After excluding duplicates and articles that did not meet the inclusion criteria, a total of 43 articles were ultimately included in our review (Figure 1). It is worth noting that most of the studies were from Taiwan, China (*N* = 29, 71%), followed by Denmark (*N* = 3, 7%), and the remaining countries were less involved (Figure 2). Data were collected over a period ranging from 1 to 13 years. In the 43 studies, 11 included the incidence of PLA, 21 on the risk factors, and 11 on the prognosis of PLA.

### 3.1. Incidence of PLA

Our research covers Asia, the Americas, Europe, and Oceania. Among these regions, the incidence of PLA is higher in Asia and the Americas. In Taiwan, China, a study encompassing one million individuals revealed that the annual incidence of PLA saw a progressive rise, increasing from 10.83 per 100,000 in 2000 to 15.45 per 100,000 in 2011 [6]. Another earlier study from the same region analyzed a total of 30,209 cases, indicating a steady increase in the annual incidence of PLA from 11.15 per 100,000 in 1996 to 17.59 per 100,000 in 2004. Meanwhile, in Korea, data spanning 2007–2017 showed an overall annual incidence of PLA at 10.9 per 100,000 for all age groups. Notably, the incidence was recorded at a mere 5.7 per 100,000 in 2007 but surged to 14.4 per 100,000 by 2017 [7]. In the United States, an analysis spanning 1994–2005 identified 17,787 PLA discharges, resulting in an overall incidence of 3.6 per 100,000. Notably, there was an annual percentage increase in the incidence of 4.1% during this period [8]. Another American study, focusing on patients aged between 1 and 20 years diagnosed with PLA, reported an incidence of 9.63 per 100,000 in 2003. By 2014, this figure had surged to 15.3 per 100,000, marking a substantial increase of nearly 60% [9]. Meanwhile, a study encompassing all Calgary Health Zone (CHZ) residents aged 20 and above with PLA found a notable rise in incidence when comparing the periods of 1999–2003 and subsequent years, with rates of 2.3 per 100,000 and 3.7 per 100,000, respectively [10, 11].

**Figure 3:**
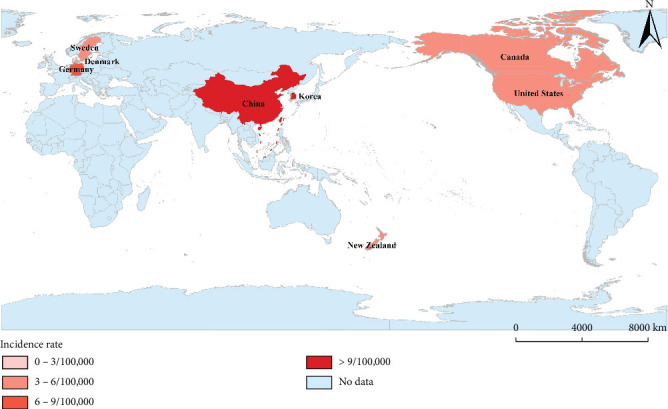
Word map showcasing the incidence of pyogenic liver abscesses across different countries.

In comparison, both Europe and Oceania have reported lower incidences of PLA. A decade-long retrospective study in Denmark identified only 52 patients meeting the PLA criteria, translating to a notably low incidence of 1.1 per 100,000 [12]. In Germany's inaugural epidemiological study on PLA, which encompassed nearly four million individuals, the incidence was determined to be 7 per 100,000, and this rate remained stable throughout the observation period [13]. Turning to Oceania, a New Zealand study retrospectively analyzed all PLA cases at Christchurch Hospital between 2014 and 2015, revealing an incidence of 5 per 100,000 [14]. In Sweden, the average incidence over the study duration stood at 3.4 per 100,000. However, there was a noticeable rise from 1.8 per 100,000 in 2011 to 5.2 per 100,000 in 2020, marking an almost threefold increase [15]. Meanwhile, Figure 3 and Supporting Information 1: Table S1 illustrate the variations in incidence rates across different regions.

### 3.2. Risk Factors for PLA

#### 3.2.1. Hepatobiliary Diseases

Liver cirrhosis stands out as a potent risk factor for PLA, often correlating with a grim prognosis. A national study conducted in Denmark revealed a high incidence of PLA in cirrhotic patients at 23.3 per 100,000. This data underscores that individuals with cirrhosis face a staggering 15-fold heightened risk of PLA compared to the general population [16]. Biliary disease, while a predominant risk factor for adults with PLA, is equally significant for pediatric cases. An American study examining PLA in children highlighted that among various comorbid conditions, hepatobiliary malignancy presented the highest odds ratio (OR 71.8). This was closely followed by liver transplant (OR 38.4) and biliary disease (OR 29.9). Notably, biliary disease amplified the risk of PLA in children by nearly sevenfold [9].

#### 3.2.2. Intestinal Disease

Intestinal disease is a risk factor of PLA. PLA is an uncommon extraintestinal complication associated with inflammatory bowel disease (IBD). A comprehensive nationwide cohort study revealed that IBD patients exhibited a marginally elevated risk of PLA compared to the control group (67.2 vs. 40.6 per 100,000), with the risk being particularly pronounced in those diagnosed with ulcerative colitis [17]. In a separate study involving 54,147 patients with diverticulosis, it was observed that individuals with diverticular disease faced a heightened risk of PLA. The incidence stood at 115 cases per 100,000 person-years, marking an incidence rate 2.44 times greater than that of the control group without diverticular disease [18].

#### 3.2.3. Diabetes Mellitus

Type 2 diabetes mellitus (T2DM) emerges as a potent risk factor for PLA, especially in patients devoid of biliary tract disease. A population-based study highlighted that the incidence of PLA in the T2DM cohort stood at 58.7 per 100,000. Intriguingly, patients newly diagnosed with T2DM faced a risk of PLA that was 2.83 times higher than their nondiabetic counterparts. Notably, younger male patients with T2DM, as well as those with a diabetes duration of less than 2 years, exhibited a heightened risk of PLA [19].

Echoing these findings, a case–control study from Denmark identified diabetes as a significant and potentially modifiable risk factor for PLA, with diabetic patients experiencing a 3.6-fold increase in PLA risk [20]. A CHZ study further reinforced this association, indicating a relative risk of 11.1 for diabetes patients in developing PLA [11]. Additionally, prior research has underscored an elevated PLA risk among male T2DM patients, particularly those under the age of 45 [21].

#### 3.2.4. Surgery

Liver transplantation is a recognized risk factor for PLA, with a national pediatric study in the United States underscoring its strong association with PLA [9]. Endoscopic sphincterotomy (ES) has also been linked to an increased risk of PLA, with an incidence of 420 per 100,000 in the ES cohort compared to 94 per 100,000 in the non-ES group (after matching for age, sex, and comorbidities), a risk persisting 5 years postprocedure [22]. A study revealed a 3.75-fold increased PLA incidence in splenectomy patients compared to nonrecipients (215 vs. 57 per 100,000), with an adjusted risk ratio of 3.89 post confounder control [23]. Similarly, appendectomy was linked to an elevated PLA risk, with a 1.73-fold higher incidence than nonrecipients (38.5 vs. 22.2 per 100,000) [24]. Research indicates that patients who underwent gastrectomy exhibited a 3.5-fold increased risk of PLA relative to controls, with incidence rates of 216 and 57.6 per 100,000, respectively [25].

#### 3.2.5. Drug-Related Factors

A case–control study involving 6407 patients revealed that current zolpidem users faced a heightened risk of PLA. Notably, the risk of PLA was dose-dependent for zolpidem, with a mean average daily dose exceeding 10 mg presenting a higher risk compared to doses ≤ 10 mg [26]. Another case–control study found that oral corticosteroid use was associated with an increased risk of PLA in adults, with a similar dose-dependent effect [27]. Research from Korea highlighted a correlation between proton pump inhibitor (PPI) use and an elevated risk of PLA [28]. Similarly, a study corroborated this association, indicating an adjusted OR of 7.59 for PPI users in relation to PLA risk [29].

#### 3.2.6. Demographic and Other Factors

Age, male gender, and lower family income have been identified as demographic risk factors for PLA, with the condition being less common in adolescents [6]. A US study emphasized that children with immunodeficiency had a threefold increased risk of PLA [9]. Kidney-related conditions have shown a strong correlation with PLA: Those with predialysis chronic kidney disease faced a 1.65-fold higher incidence, and end-stage renal disease patients had an incidence of 182 per 100,000 compared to 63.4 per 100,000 in controls [30, 31]. Chronic pancreatitis (CP) patients, especially those with multiple comorbidities, also exhibited a heightened risk, with an incidence 12.9 times higher than non-CP individuals [32]. Other significant risk factors include pneumonia, periodontitis in adults under 50 years of age, herpes zoster [33–35], and alcohol intoxication, with the latter group having a 3.47-fold higher incidence of liver abscess [36]. Further details on the risk factors associated with PLA are illustrated in Supporting Information 2: Table S2.

### 3.3. Subsequent Health Outcomes

#### 3.3.1. Gastrointestinal Tumor

A population-based study found that patients with PLA faced a 4.30-fold increased risk of gastrointestinal cancer, suggesting PLA might be an early indicator of this cancer type. Notably, the incidence rate was 1080 per 100,000 for PLA patients compared to 251 per 100,000 for controls. Within this cohort, colorectal cancer was most prevalent, but there were also significant risks associated with small intestine, biliary tract, and pancreatic cancers [37]. Another study emphasized the heightened risk of primary liver cancer (PLC) following a PLA diagnosis. The incidence of PLC in PLA patients was nearly double that of controls, with specific increases observed for hepatocellular carcinoma (1.5-fold) and a striking 11-fold rise for intrahepatic cholangiocarcinoma. This research underscores the particular vulnerability of PLA patients to intrahepatic cholangiocarcinoma [38]. Further supporting this association, another study identified PLA as a potential warning sign for PLC, with PLA patients having a 3.4-fold higher hazard ratio for PLC compared to controls [39].

#### 3.3.2. Infection

PLA has been linked to an elevated risk of subsequent infections. Notably, there was a pronounced risk for splenic and perinephric abscesses [40]. A nationwide study highlighted the heightened risk of pneumonia within 90 days post-PLA diagnosis. The incidence rates were 95.9 per 100,000 for PLA patients and 18.7 per 100,000 for controls. After adjusting for confounders, PLA patients had a 5.28-fold increased hazard ratio for pneumonia [41]. Furthermore, PLA patients also faced a significantly elevated risk of prostatic abscess, with occurrences in 0.330% of PLA patients compared to 0.010% in non-PLA patients [42]. Another study pinpointed an increased risk of endophthalmitis among PLA patients, identifying the condition in 106 out of 12,727 subjects [43].

#### 3.3.3. Other Diseases

**Figure 4:**
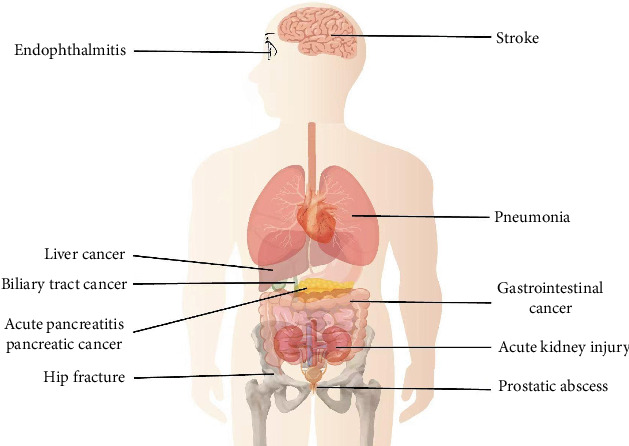
Conceptual diagram highlighting the impact of pyogenic liver abscesses on subsequent health outcomes.

A comprehensive study highlighted that PLA patients faced a 1.17-fold elevated risk of hip fractures compared to controls. Notably, the risk surged with the frequency of hospital admissions: PLA patients admitted two to three times annually had an 18.4-fold increased risk, and those admitted four or more times faced a staggering 46.0-fold risk; the study also found a higher risk of hip fracture in patients aged 45–64 years and in female subjects. However, the impact of PLA on the occurrence of hip fracture was more prominent among subjects aged < 45 years old [44]. Neurological implications of PLA have also been observed. A study after follow-up found slightly higher stroke rates at 3, 6, and 12 months in PLA patients than in non-PLA patients (2.15% vs. 1.10%, 3.20% vs. 1.91%, and 4.76% vs. 3.43%, respectively) [45]. Additionally, PLA has been linked to an elevated risk of acute pancreatitis, with the overall incidence of acute pancreatitis being 3.84-fold greater in the PLA group than in the non-PLA group, and it was found that the increased risk in the PLA group remained for the 5-year follow-up, especially within the initial 3 months postdiagnosis [46]. In terms of renal health, a large-scale study associated PLA with a heightened risk of acute kidney injury (AKI). The PLA cohort had a 1.51-fold higher AKI incidence than controls. Even after adjusting for confounders, the AKI risk for PLA patients remained elevated, especially for those without other comorbidities [47]. Further details on the subsequent health outcomes associated with PLA are illustrated in Figure 4 and Supporting Information 3: Table S3.

## 4. Discussion

To our knowledge, this is the first review exploring the extent of PLA, including incidence, risk factors, and subsequent health outcomes. It is worth noting that this scoping review, based on large population sample data, is more representative and avoids the specificity of results obtained from small sample data. We found that the incidence of PLA was generally increasing worldwide, which is consistent with previous reports [48–50]. This may be related to improvements in diagnostic methods or changes in the definition of an abscess.

The incidence of PLA varies between different areas. The incidence in Asia was the highest in the current study, probably because of the transmission of pathogenic clones of *K. pneumoniae* [51]. In the field of microbiology, the genera *Escherichia coli* and *Streptococcus* are the most common microorganisms associated with PLA in western countries, while *K. pneumoniae* and *E. coli* dominate in Asia [52]. The emergence of hvKP and its worldwide spread may also be a factor in the increasing incidence of PLA [53]. In addition, many PLA patients from Europe and America are also Asian [54]. Moreover, this altered etiology with *K. pneumoniae* as the main pathogen may also account for the increased risk of infection after PLA and may increase the risk of bacteraemia and metastatic complications [40, 55, 56].

Some studies have also reported a high incidence in rural areas [49, 57], suggesting that the incidence of PLA may be related to the degree of economic development, but no studies with large samples have confirmed this.

Intestinal diseases are the main cause of PLA in portal vein route infection, while damage to the intestinal mucosa is the main cause of pathogenic bacterial invasion, of which *K. pneumoniae* is currently reported to be the most important pathogenic bacterium [58]. Hepatic artery thrombosis (HAT) is a serious complication that arises after liver transplantation. It can cause liver and biliary ischemia, induce bile duct lesions, and promote the formation of abscesses [58]. According to a retrospective study, the incidence of liver abscesses after liver transplantation is 480/100,000 transplant patient-years [58]. Moreover, biliary stricture, diabetes mellitus, and Roux-en-Y choledochojejunostomy are also the risk factors for PLA after liver transplantation [58]. Therefore, close clinical monitoring of liver transplant recipients may help reduce the incidence of PLA.

Patients with PLA have a significantly higher risk of gastrointestinal tumors, especially colorectal cancer, compared with the general population. This phenomenon may be associated with intestinal dysbiosis and oxidative stress. Additionally, colorectal cancer can disrupt the colonic mucosal barrier, creating a pathway for bacterial translocation into the portal vein system. This suggests that undiagnosed colorectal cancer is a potential underlying etiology of cryptogenic PLA. In clinical practice, most patients with cryptogenic PLA undergo colonoscopy following diagnosis, which significantly enhances the detection rate of colorectal cancer. A US study showed that the risk of colorectal cancer was significantly elevated in PLA patients in the first 3 years after diagnosis of PLA. However, after 3 years, this risk was no longer significant [59]. At present, the specific link between PLA and gastrointestinal tumors cannot be defined, and further research is still required to clarify this relationship. It is worth noting that doctors should consider the possibility of screening gastroscopy for gastrointestinal tumors when dealing with patients with PLA in clinical practice.

Diabetic patients have decreased immunity, thus their anti-infection ability is poor, as the high blood sugar level will inhibit the function of white blood cells, while also further promoting the growth of bacteria, inducing the formation of PLA [48, 60]. Meanwhile, we found that diabetes not only is a high risk factor for PLA but also is more likely to result in a poor prognosis [61]. Therefore, strict blood glucose control is an important strategy to reduce the occurrence of PLA [62]. The use of a PPI will also increase the incidence of PLA, as it affects intestinal microbes and also suppresses the immune killing ability of neutrophils. Therefore, the indications for the use of PPIs should be strictly controlled in clinical practice.

Most articles focused on the subsequent effects of cancer and infection. Studies have shown that the incidence of colorectal cancer in patients with PLA is 2.68 times higher than that in the general population [63]. PLA may be an early marker for colorectal cancer. So some studies suggest that for patients with cryptogenic PLA, colorectal cancer screening should be performed [64]. PLA patients are also at an increased risk of other infections, which may be related to impaired immune function. Meanwhile, the effect of PLA on fracture and cardiovascular and cerebrovascular diseases also deserves attention, but the specific reasons need to be further studied.

## 5. Limitations

Our study undoubtedly has some limitations. First, although we aimed to provide a comprehensive analysis, the current number of studies related to PLA is relatively small, which may compromise the generalizability of the results. Second, the data involved in this study span a large period (1977–2020), during which the diagnostic criteria and medical practices changed in many countries. So, the early incidence of PLA may have been underestimated. Finally, given that most of the studies are derived from the early stage and are in the context of population aging, it is expected that more multicenter and regional studies on PLA will be conducted in the future.

## 6. Conclusion

Taiwan, China, emerges as the focal point of population-based research on PLA, with consistent findings indicating a year-on-year rise in PLA incidence. Key risk factors identified include liver cirrhosis, hepatobiliary malignancy, liver transplantation, biliary disease, and diabetes mellitus. Furthermore, a PLA diagnosis significantly elevates the risk of subsequent health complications. This encompasses a broad spectrum of conditions, from gastrointestinal tumors to infections. The increasing prevalence and multifaceted implications of PLA underscore the need for medical professionals to remain updated on its epidemiology, risk factors, and subsequent health outcomes. Such awareness is pivotal for effective community prevention, clinical intervention, and long-term patient management.

## Data Availability

The authors have nothing to report.

## Nomenclature

PLA: pyogenic liver abscess
CNKI: China National Knowledge Infrastructure
KPLA: *Klebsiella pneumoniae* liver abscess
hvKP: hypervirulent *Klebsiella pneumoniae*
PRISMA-ScR: Preferred Reporting Items for Systematic Reviews and Meta-Analyses Extension for Scoping Reviews
CHZ: Calgary Health Zone
IBD: inflammatory bowel disease
T2DM: Type 2 diabetes mellitus
ES: endoscopic sphincterotomy
PPI: proton pump inhibitor
CP: chronic pancreatitis
PLC: primary liver cancer
AKI: acute kidney injury
HAT: hepatic artery thrombosis

## Supporting Information

Supporting Information 1 Additional supporting information can be found online in the Supporting Information section. Table S1: Incidence and risk factors associated with PLA in different countries and regions.

Supporting Information 2 Table S2: Incidence and risk factors associated with PLA.

Supporting Information 3 Table S3: Overview of the impact of PLA on subsequent health outcomes.
