# Supplementary material for: Incidence, Risk Factors, and Subsequent Health Outcomes of Pyogenic Liver Abscesses: A Scoping Review of Evidence From Population-Based Studies
Source: Gastroenterol Res Pract. 2025 Sep 23;2025:3915024. doi: 10.1155/grp/3915024 (PMC12483727; doi:10.1155/grp/3915024)
Supplement: Supporting Information 1 — Additional supporting information can be found online in the Supporting Information section. Table S1: Incidence and risk factors associated with PLA in different countries and regions. [file 3915024.f1.docx]

**Supplementary Table 1 Incidence and Risk Factors Associated with PLA in Different Countries and Regions**

| Study | Country or region | Year of Study | Study population size | Incidence | Risk factors |
| --- | --- | --- | --- | --- | --- |
| Chen YC et al.(6) | Taiwan, China | 2000-2011 | 1,000,000 | 10.83/100,000 to 15.45/100,000 | Male; patients aged >50 years; lower income |
| Tsai FC et al.(7) | Taiwan, China | 1996-2004 | 30,209 | 11.15/100,000 in 1996 to 17.59/100,000 in 2004 | Diabetes mellitus; |
| Yoo JJ et al.(8) | Korea | 2007-2017 | 30,690 | 5.7/100,000 to 14.4/100,000 | Diabetes mellitus |
| Meddings L et al.(9) | America | 1994-2005 | 17,787 | 3.6/100,000 | Hepato-biliary disease; diabetes mellitus; liver transplantation |
| Thavamani A et al.(10) | America | 2003-2014 | 44,486 | 9.63/100,000 to 15.3/100,000 | liver transplantation; biliary disease; immunodeficiency; appendicitis |
| Losie JA et al.(11) | Calgary Health Zone | 2015-2017 | 1,300,000 | 3.7/100,000 | Male; liver transplantation; diabetes mellitus; alcohol use disorder; |
| Kaplan GG et al.(12) | Calgary Health Zone | 1994-2003 | About 1,000,000 | 2.3/100,000 | Liver transplantation; diabetics; male; older patients; diabetes mellitus |
| Hansen PS et al.(13) | Denmark | 1986-1996 | Not mentioned | 1.1/100,000 | Diabetes mellitus |
| Zimmermann L et al.(14) | Germany | 2013-2019 | 4,000,000 | 7/100,000 | Intestinal and biliary diseases; liver transplantation |
| Kubovy J et al.(15) | New Zealand | 2014-2015 | Not mentioned | 5/100,000 | Diabetes mellitus |
| Svensson E et al.(16) | Sweden | 2011-2020 | 1,400,000 | 1.8/100,000 to 5.2/100,000 | Not mentioned |
