# Supplementary material for: Incidence, Risk Factors, and Subsequent Health Outcomes of Pyogenic Liver Abscesses: A Scoping Review of Evidence From Population-Based Studies
Source: Gastroenterol Res Pract. 2025 Sep 23;2025:3915024. doi: 10.1155/grp/3915024 (PMC12483727; doi:10.1155/grp/3915024)
Supplement: Supporting Information 2 — Table S2: Incidence and risk factors associated with PLA. [file 3915024.f2.docx]

**Supplementary Table 2 Incidence and Risk Factors Associated with PLA**

| Study | Country or region | Year of Study | Study population size | Incidence | Odds ratio, Hazard ratio, or Relative risk | Risk factors |
| --- | --- | --- | --- | --- | --- | --- |
| Mølle I et al.(17) | Denmark | 1977-1993 | 23,429 | 23.3/100,000 | Not mentioned | Liver cirrhosis |
| Lin JN et al.(18) | Taiwan, China | 2000-2010 | 57,520 | 67.2 vs 40.6/100,000 | SHR=1.46 | Inflammatory bowel disease |
| Tsai MS et al.(19) | Taiwan, China | 1998-2010 | 270,735 | 115 vs 46.5/100,000 | aHR=2.11 | Colonic diverticular diseases |
| Wang TY et al.(20) | Taiwan, China | 2000-2011 | 223,640 | 58.7 vs 20.6/100,000 | HR=2.83 | Diabetes mellitus;  male;  gallstone and cholangitis |
| Thomsen RW et al.(21) | Denmark | 1977-2002 | 73,780 | Not mentioned | aRR=3.60 | Diabetes mellitus;  benign biliary obstruction;  cancer; ongoing abdominal infection |
| Ko MC et al.(22) | Taiwan, China | 2000-2010 | 1,228,534 | 115.40 vs 36.58/100,000 | HR=2.88 | Type 2 diabetes mellitus;  biliary tract diseases;  liver cirrhosis |
| Peng YC et al.(23) | Taiwan, China | 2000-2010 | 16,348 | 420 vs 94/100,000 | aHR=4.50 | Endoscopic sphincterotomy |
| Lai SW et al.(24) | Taiwan, China | 1998-2010 | 88,634 | 215 vs 57/100,000 | aHR=3.89 | Splenectomy |
| Liao KF et al.(25) | Taiwan, China | 1998-2010 | 1,062,629 | 38.5 vs 22.2/100,000 | HR=1.77 | Appendectomy |
| Tsai MS et al.(26) | Taiwan, China | 2000-2010 | 169,170 | 216 vs 57.6/100,000 | aHR=3.08 | Gastrectomy |
| Liao KF et al.(27) | Taiwan, China | 2000-2011 | 6,407 | Not mentioned | OR=3.89 | Zolpidem use |
| Lai SW et al.(28) | Taiwan, China | 2000-2013 | 4,088 | 20.1% vs 14.8% (rate) | aOR=1.40 | Oral corticosteroid use |
| Oh JH et al.(29) | Korea | 2003-2013 | 4,209,229 | 88 vs 13/100,000 | aHR=4.19 | Proton pump inhibitor use |
| Lin HF et al.(30) | Taiwan, China | 2000-2011 | 2,744 | Not mentioned | aOR=7.59 | Proton pump inhibitor use |
| Lai SW et al.(31) | Taiwan, China | 2000-2010 | 162,236 | 138 vs 83/100,000 | HR=1.51 | Predialysis chronic kidney disease; biliary stone;  chronic liver disease;  diabetes mellitus |
| Tsai LW et al.(32) | Taiwan, China | 1998-2006 | 115,522 | 182 vs 63.4/100,000 | HR=3.63 | End-stage renal disease;  diabetes mellitus;  old age; cirrhosis; hypertension |
| Tseng CW et al.(33) | Taiwan, China | 2000-2011 | 89,050 | 3830 vs 289/100,000 | aHR=6.40 | Chronic pancreatitis |
| Yeh YT et al.(34) | Taiwan, China | 2009-2013 | 3,455 | 26.52% vs 15.53% (rate) | aOR=2.31 | Periodontitis |
| Ho SW et al.(35) | Taiwan, China | 2010-2013 | 2,470 | Not mentioned | aOR=2.104 | Pneumonia; biliary stone |
| Mei-Ling S(36) | Taiwan, China | 1998-2010 | 164,756 | 44.7 vs 32.5/100,000 | aHR=1.34 | Herpes zoster |
| Wang YC et al.(37) | Taiwan, China | 2000-2010 | 306,345 | 122 vs 34.3/100,000 | aHR=2.64 | Alcohol intoxication |
