# Supplementary material for: Incidence, Risk Factors, and Subsequent Health Outcomes of Pyogenic Liver Abscesses: A Scoping Review of Evidence From Population-Based Studies
Source: Gastroenterol Res Pract. 2025 Sep 23;2025:3915024. doi: 10.1155/grp/3915024 (PMC12483727; doi:10.1155/grp/3915024)
Supplement: Supporting Information 3 — Table S3: Overview of the impact of PLA on subsequent health outcomes. [file 3915024.f3.docx]

**Supplementary Table 3 Overview of the Impact of PLA on Subsequent Health Outcomes**

| Study | Country or region | Year of Study | Study population size | Subsequent health outcomes |
| --- | --- | --- | --- | --- |
| Lai HC et al.(38) | Taiwan, China | 2000-2007 | 73,450 | Gastrointestinal cancer (1080 per 100,000 person-years） |
| Chu CS et al.(39) | Taiwan, China | 2000-2008 | 87,655 | Delayed-onset primary liver cancer (293 per 100,000 person-years) |
| Huang WK et al.(40) | Taiwan, China | 2000-2008 | 3,974 | Primary liver cancer (601.5 per 100,000 person-years) |
| Keller JJ et al.(41) | Taiwan, China | 2006-2008 | 72,300 | Infections |
| Chung SD et al.(42) | Taiwan, China | 2006-2008 | 77,208 | Pneumonia (95.9 per 100,000 person-days) |
| Chung SD et al.(43) | Taiwan, China | 2006-2008 | 86,625 | Prostatic abscess |
| Hu CC et al.(44) | Taiwan, China | 2006-2008 | 76,362 | Endophthalmitis |
| Hsu CC et al.(46) | Taiwan, China | 1999-2010 | 127,650 | Hip fracture (448 per 100,000 person-years) |
| Keller JJ et al.(47) | Taiwan, China | 2006-2008 | 59,862 | Stroke (4.76%） |
| Lai SW et al.(48) | Taiwan, China | 2000-2010 | 154,330 | Acute pancreatitis (461 per 100,000 person-years) |
| Sung CC et al.(49) | Taiwan, China | 2000-2011 | 159,435 | Acute kidney injury(925 per 100,000 person-years) |
|  |  |  |  |  |
